# The transition between M1 and M2 macrophage phenotypes is associated with the disease status following CD19 CAR-T therapy for B cell lymphoma/leukemia

**DOI:** 10.1038/s41419-025-07610-3

**Published:** 2025-04-11

**Authors:** Li Zhao, Fen Yan, Donghai Tang, Wenwen Li, Na An, Chunxiao Ren, Ying Wang, Kailin Xu, Kai Zhao

**Affiliations:** 1 Department of hematology, The Affiliated Hospital of Xuzhou Medical University, Xuzhou, Jiangsu China; 2 Blood diseases Institute, Xuzhou Medical University, Xuzhou, Jiangsu China

**Keywords:** Cancer immunotherapy, Haematological cancer

## Abstract

Although anti-CD19 chimeric antigen receptor (CAR-T) cells demonstrate high response rates in relapsed/refractory B-cell lymphomas, a considerable proportion of patients eventually encounter disease progression or relapse. The short-term and long-term outcomes of CAR-T treatment are intricately linked to the tumor microenvironment (TME), wherein macrophages with polarized characteristics can exhibit either anti-tumorigenic or pro-tumorigenic roles. Despite evidence implicating the crucial involvement of macrophages in CAR-T cell-treated lymphoma, their dynamic distribution and immune function related to lymphoma progression remain poorly understood. Immunocompetent mice were utilized to establish syngeneic A20 lymphoma/leukemia models. The distribution and polarization of macrophages were detected using immunohistochemistry (IHC) and flow cytometry techniques. We observed that CD19 CAR-T therapy exhibited significant efficacy in protecting mice against lymphoma, leading to increased infiltration of macrophages into the tumor tissue. Notably, during remission stages, M1-like macrophages (CD11b^+^F4/80^+^C206^−^CD80^+^) were predominant, whereas in relapsed mice, there was a shift towards M2-like phenotypes (CD11b^+^F4/80^+^C206^+^CD80^+^). The transition from remissive to relapsed status was accompanied by a reduction in the M1/M2 ratio and a decrease in pro-inflammatory cytokines. Furthermore, quantitative real-time polymerase chain reaction (qRT-PCR) analysis confirmed differential expression levels of CD206 and CD163 between remissive and relapsed mice, while signaling pathways involving PI3K and STAT3 may contribute to the skewing towards M2 polarization. In summary, our findings highlight the dynamic transformation of macrophage polarization during different stages of lymphoma progression and underscore its potential implications for immunotherapeutic interventions.

## Introduction

Chimeric antigen receptor (CAR) T cell therapy targeting CD19 has demonstrated efficacy in the treatment of refractory and relapsed B cell lymphomas, with complete response (CR) rates ranging from 39% to 88% [1–5]. However, a significant proportion of patients treated with CD19 CAR-T-cell eventually experience disease progression or relapse. In the ZUMA-1 trial, more than half of the patients with advanced large B-cell lymphoma (LBCL) exhibited disease progression at a median follow-up duration of 15.4 months, while the median overall survival (OS) was reported as 25.8 months [2, 6–8]. In a 3-year investigation of JULIET, 71 of 115 patients had disease progression or died on study [9]. The outcomes of CAR-T treated patients were not only independent of the depth of initial response, expansion, and persistence of CAR-T cells, but also influenced by the tumor microenvironment (TME) as co-variates [10]. TME plays a pivotal role in modulating the efficacy of CAR-T cell therapy for lymphoma, during which the infusion of CAR-T cells elicits a broad activation of the host immune system [11, 12]. Macrophages constitute the predominant population of innate immune cells within the TME, exerting a profound influence on the efficacy of immunotherapy [13]. Evidence shows that macrophage numbers rapidly increase as early as 18 h after CAR-T administration, with macrophages emerging as the main source of IL-6 and iNOS at the site of CAR-T cell-tumor tissue co-localization, inducing cytokine release syndrome [14]. However, the functional and status characteristics of macrophages in the TME during early-stage remission and late-stage disease relapse or resistance to CAR-T therapy remain poorly understood.

The pathophysiological adaptations of macrophages are intricately intertwined with disease progression. Tumor-associated macrophages (TAMs), accounting for ~50% of hematopoietic cells in the TME, exhibit heterogeneous properties ranging from anti-tumorigenic to pro-tumorigenic [15]. Anti-tumorigenic TAMs known as M1-like type retain properties of high expression of MHC II, phagocytosis, tumor-killing activity, and secrete pro-inflammatory cytokines that support and activate adaptive immune cells. Conversely, pro-tumorigenic TAMs with M2-like phenotypes support cancer cell growth and mediate immunosuppressive effects on the adaptive immune cells of the TME [16, 17]. In a phase I clinical trial evaluating the efficacy of CD19 CAR-T cells in refractory B-cell non-Hodgkin’s lymphoma (B-NHL), increased infiltration of TAMs with M2 phenotype was found to be negatively associated with remission status [18]. In murine models of B-lymphoma, we observed that upon achieving remission after CD19 CAR-T treatment, F4/80^+^ macrophages exhibited enhanced MHC II and CD80 expression in the bone marrow (BM), spleen (SP), and liver compared to mice without CD19 CAR-T administration. However, a decline in myeloid cell activation was evident when lymphoma mice experienced relapse [19]. Regarding the underlying mechanisms of macrophage polarization switch, studies have shown that in B-lymphoma cells, CREBBP/EP300 mutations inhibit H3K27 acetylation, leading to decreased FBXW7 expression, activation of the NOTCH pathway, and subsequent upregulation of CCL2/CSF1 expression. This cascade ultimately results in TAM polarization towards the M2 phenotype and promotes tumor cell proliferation [20]. Analysis of key immune pathways associated with CR versus non-CR in two independent datasets (ZUMA-1 and Axi-cel patients) revealed that genes related to antigen processing and presentation, particularly those associated with M2 macrophages and myeloid activation, exhibited a significant decrease in fold change [21]. Additionally, increased cholesterol efflux from M2 macrophages in DLBCL patients with progressive disease was found to inhibit CD19 CAR-T cell cytotoxicity by inducing CD8^+^ T cell exhaustion [22]. In summary, the aforementioned data suggest a close association between the status and function of macrophages and the lymphoma progression following CD19 CAR-T therapy. As CAR-T cells continue to gain widespread usage, comprehending the status and function of macrophages in the TME post-CAR-T therapy and overcoming their immunosuppressive barriers will be pivotal in fully harnessing the potential of CAR-T as an exceptionally effective treatment modality.

Macrophages might exhibit a dichotomous nature with the potential to exert both pro- and anti-tumorigenic effects, which is indicative of their adaptability in response to diverse environmental stimuli following CAR-T therapy. In this study, we conducted an analysis of TAMs in an immunocompetent A20-seeded lymphoma/leukemia murine model following administration of CD19 CAR-T cells. Our findings suggest that TAM transformation occurs during CAR-T treatment, and the ratio of M1/M2 polarization bias within the TME may serve as a potential indicator for disease progression.

## Materials and methods

### Mice

Female BALB/c mice, aged 6–8 weeks, were procured from Beijing Viton Lever. The mice were housed in a pathogen-free facility at Xuzhou Medical University. All mice were uniquely identified using ear tags and randomly allocated to different experimental groups via a random number table method after determining the sample size based on preliminary experiment results. Furthermore, during the stages of mouse modeling, treatment, and analysis of experimental outcomes, the experimenters were blinded to the group assignments. All mice were included in the experiments, with utmost efforts made to minimize animal suffering. All experimental procedures conducted in this study adhered to the approved protocol by the Ethics Committee for experimental animals.

### Retrovirus package and preparation of murine CD19 CAR-T cells

The plasmid of anti-mouse CD19 CAR was kindly provided by Prof. Peng Li who is from Guangzhou Institute of Biomedicine and Health, Chinese Academy of Science. Fifteen micrograms of plasmids and 45 μl of X-treme GENE 9 DNA transfection reagent(Roche) were transfected into Plat-E cells (ATCC). The cell supernatant containing the retrovirus was collected at 24, 48, and 72 h of transfection and stored at 4 °C after filtering through 0.45 μm filters. Splenic CD3^+^ T cells from BALB/c mice were isolated and purified using EasySep negative selection reagents according to the manufacturer’s instructions (StemCell Technologies). Purified T cells (2 × 10^6^/mL) were activated in a 24-well plate pre-coated with anti-CD3 mAb (2 mg/ml, BioLegend) and soluble anti-CD28 mAb (2 μg/mL, BioLegend) for 24 h or 36 h, and then transfected with packaged retroviruses in the present of IL-2 for 72 h. Finally, infected T cells were collected, and the percentage of GFP^+^ CAR T cells was detected by flow cytometry.

### Establishment and evaluation of murine model of local B cell lymphoma

The A20 lymphoma cell line (stored in our laboratory) was cultured in RPMI-1640 medium supplemented with β-mercaptoethanol and incubated at 37 °C in a 5% CO_2_ incubator. The Matrigel^TM^ matrix gel and PBS were mixed in a 1:1 ratio, followed by resuspension of A20 cells at a concentration of 1 × 10^7^/mL. Subsequently, we injected BALB/c mice with 200 μL of the prepared cell suspension into the right abdomen on day—7. On day 0, either 1 × 10^6^ mCD19 CAR-T cells (remission group) or activated T cells (control group) were administered. To establish the relapse model of local lymphoma after CAR-T treatment, A20 cells (1 × 10^6^/mouse) were subcutaneously injected on day—14, and intraperitoneal administration of cyclophosphamide at a dose of 80 mg/kg was performed on day—1. Subsequently, mCD19 CAR-T cells were infused on day 0. The long and short diameters of the tumors were measured every 2 days, and their volume was calculated using the formula V = a × b^2^/2. The criteria for remission and relapse were detailed in Supplementary Fig. 1.

### Establishment and evaluation of murine model of systemic B cell lymphoma/leukemia

BALB/C mice were irradiated with a dose of 3.5 Gy on day—3, followed by intravenous injection of A20-luciferase cells (1 × 10^6^/mouse) after 4 h. On day 0, mCD19 CAR-T cells (2 × 10^5^/mouse) were infused, while a control group receiving anti-human CD19 CAR-T cell infusion was established simultaneously. The mice were monitored daily for survival and every 3 days for changes in body weight. In accordance with our previous publication [14], the clinical scores were evaluated based on 3 parameters: weight loss, activity level, and paralysis. Each parameter was assigned a severity scale, with a maximum score of 15. The remission of mice was defined as the period during which the clinical score remained at 0, while the relapse of mice was defined as the observation of a clinical score greater than 1. The experimental design and criteria for clinical scoring are elaborated in Supplementary Fig. 2. The IVIS Lumina S5 system (Shenzhen Myriad BioMedical Co., Ltd) was employed for in vivo imaging to assess tumor burden in mice, with fluorescence intensity of the tumor observed on days 5 and 12, respectively.

### Cell extractions

The subcutaneous tumor, BM, SP, and liver were collected from mice during the periods of remission and relapse. Tumor tissue and SP were mechanically dissociated, and single cell suspensions were prepared by passing the cells through a 200 μM cell strainer. BM cells were isolated by flushing the femur and tibias. Livers were minced, followed by centrifugation at 400 rpm for 3 min at 4 °C to remove non-hematopoietic cells. The supernatant was then transferred to a new tube and further centrifuged at 1500 rpm for 10 min. The cell pellet was re-suspended in RPMI-1640 medium, and lymphocytes were subsequently isolated using a 25% Optiprep gradient (StemCell).

### Flow cytometry and antibodies

Single cell suspensions from tumor tissue, BM, SP and liver were stained using the following mAb from BioLegend: CD3-FITC (Cat: 100204), CD19-PE/Dazzle™ 594 (Cat: 115553), CD4-PE-CY7 (Cat: 100422), CD11b-BV421 (Cat: 101251), F4/80-BV510 (Cat: 123135), IL-6-PE (Cat: 504504), IL-10-PE/Dazzle™594 (Cat: 505034), IL-12-PE-CY7(Cat: 505210), TNF-α-APC(Cat: 506308); and Elabscience CD3-eV450 (Cat: E-AB-F1013UQ), CD80-PE (Cat: E-AB-F0992UD), CD206-APC (Cat: E-AB-F1135UE), CD86-PE-CY7 (Cat: E-AB-F0994UH). For intracellular cytokine staining, cells were stimulated with PMA (50 ng/mL, Sigma) and Ionomycine (750 ng/ mL, Sigma) in the presence of Brefeldin A (10 mg/ mL, Invitrogen) for 4 h at 37°C. Flow cytometry was performed using a BD LSRFortessa Fortessa flow cytometer (RRID: SCR_019601). Data were analyzed using FlowJo software (FlowJo X, BD Inc. RRID: SCR_008520).

### Immunofluorescence

Tumor tissues combined with adjacent tissues and femurs were collected during remission and relapse stages, followed by fixation in 4% paraformaldehyde at 4 °C for 24 h. Subsequently, the samples were embedded in paraffin blocks and sectioned into 5 μM-thick slices. After dewaxing, hydration, and antigen retrieval steps, these sections were initially incubated in dark for 25 min with a solution of 3% hydrogen peroxide. Then, they were blocked with 3% bovine serum albumin for another 30 min at room temperature before being incubated overnight at 4 °C with EMR1 pAb (27044-1-AP, Proteintech) and Anti-CD206 pAb (GB113497, rabbit, Servicebio). After washing the sections three times with PBS, the sections were initially incubated with HRP-conjugated Goat Anti-Rabbit IgG (H+L) (GB23303, Servicebio) for 50 min. Subsequently, they were incubated with iF488-Tyramide (G1231, Servicebio) and CY3-Tyramide (G1223, Servicebio) for 10 min under light-protected conditions. Finally, nuclei were stained with DAPI and images were captured using an orthogonal fluorescence microscope (Nikon Eclipse C1, Nikon, Japan) and a scanner (Pannoramic MIDI, 3DHISTECH). Quantitative fluorescence analysis was performed using Image J software.

### qRT-PCR

The total RNA was extracted from the samples using Trizol (Cat:15596018; Ambion), and the quantity and quality of RNA were assessed with a NanoDrop 2000 (Thermo Scientific, USA) following the manufacturer’s instructions. Subsequently, reverse transcription to cDNA was performed, followed by qPCR using RT Master Mix for qPCR II (Cat: HY-K0510A; MCE) and Magic SYBR Mixture (Cat: CW3008M; CWBIO) according to the manufacturer’s protocols. Melting curve analysis was conducted to evaluate PCR product specificity. The primer sequences are provided in Table 1. The expression levels of target genes were normalized against GAPDH, and relative expression was quantified using the 2^−ΔΔct^ method.

**Table 1:** List of primer sequences used in this study.

Gene symbol	Forward(5′-3′)	Reverse(5′-3′)
GAPDH	TGTTCGTCATGGGTGTGAAC	ATGGCATGGACTGTGGTCAT
CD206	GAGCCTGGAAAGAGCTGTGT	ACCCTCCGGTACTACAGCAT
CD163	GGTGGACACAGAATGGTTCTTC	CCAGGAGCGTTAGTGACAGC
Arg1	CTCCAAGCCAAAGTCCTTAGAG	AGGAGCTGTCATTAGGGACATC
NOS2	GCCAACATGCTACTGGAGGT	GCAAAGAGGACTGTGGCTCT
IL-4	CTCAACCCCCAGCTAGTTGT	TGCATGATGCTCTTTAGGCT
IL-10	ATCTCCCTGGTTTCTCTTCCC	CCCTTTGCTATGGTGTCCTTTC
TNF-α	GACGTGGAACTGGCAGAAGAG	TTGGTGGTTTGTGAGTGTGAG
TGF-β	CTCCCGTGGCTTCTAGTGC	GCCTTAGTTTGGACAGGATCTG
PI3K	TGTGGCACAGACTTGGTGTT	TTCTTCCCTTGAGATGTCTCCC
STAT1	GCTGCCTATGATGTCTCGTTT	TGCTTTTCCGTATGTTGTGCT
STAT3	CAATACCATTGACCTGCCGAT	GAGCGACTCAAACTGCCCT
STAT6	CTCTGTGGGGCCTAATTTCCA	CATCTGAACCGACCAGGAACT

### Statistical analysis

Statistical analyses were conducted following the exclusion of outliers identified through the box plot method. If the variance was chi-square, comparisons between two groups were performed using Student’s *t* test, and multi-group comparisons were analyzed by one-way or two-way ANOVA; if the variance was not chi-square, nonparametric tests were used. Survival rates were compared using the log-rank test. Flow plots were generated using FlowJo Software v10.6.2. Statistical analyses and graphs were performed using GraphPad Prism 9 software (GraphPad Prism, San Diego, RRID: SCR_002798). *P*-values < 0.05 were considered statistically significant.

## Results

### Macrophages involved in the course of mCD19 CAR-T therapy against lymphoma

**Fig. 1:**
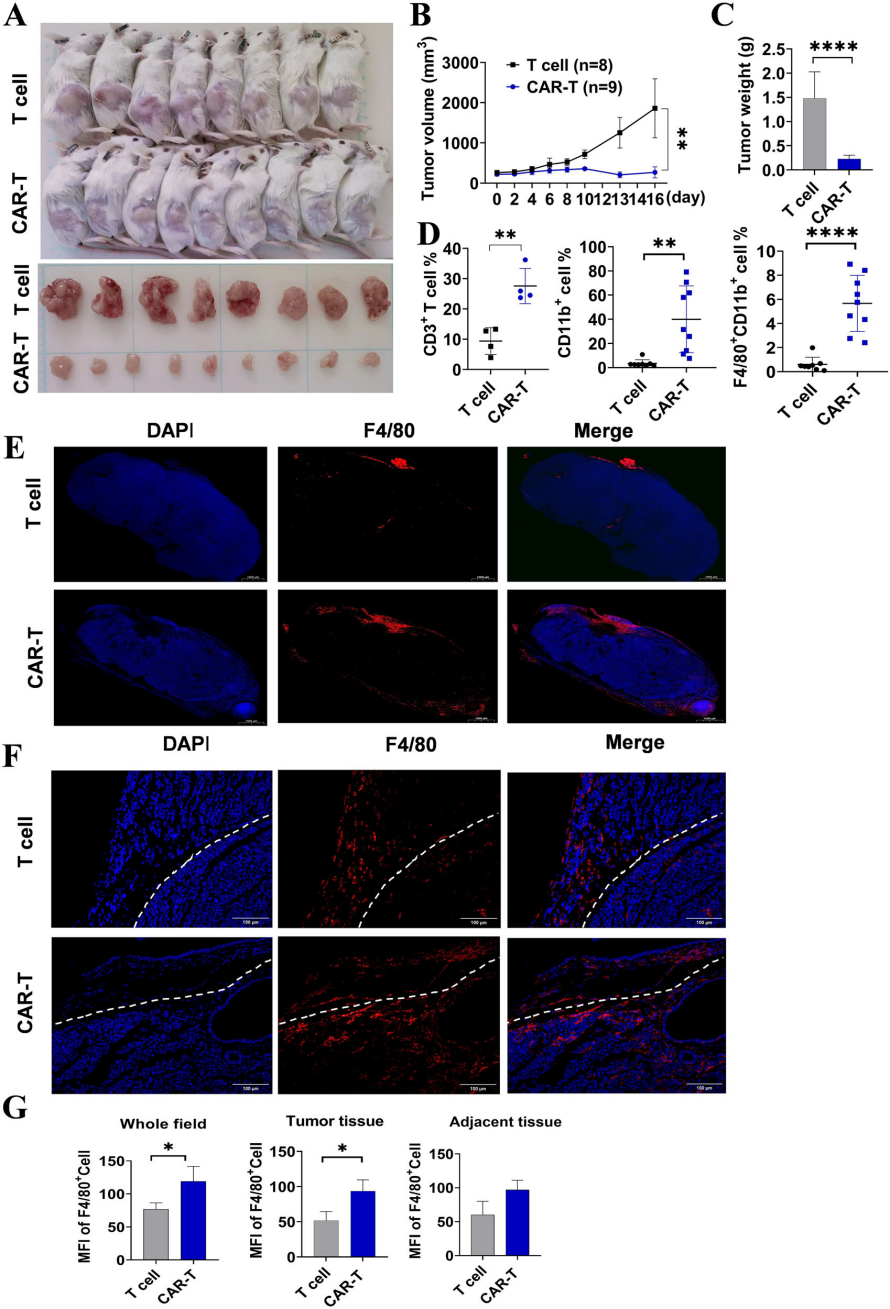
The efficacy of mCD19 CAR-T cell on subcutaneous lymphoma and the distribution of macrophages in TME. **A** Subcutaneously seeded lymphoma in mice with or without CD19 CAR-T cell treatment. **B** The changes in tumor volume were quantified and subjected to statistical analysis. Statistical analyses were performed using the two-way ANOVA. **C** Tumors were excised and their weights were recorded. **D** Proportions of CD3^+^ T cells, CD11b^+^ myeloid cells, and F4/80^+^CD11b^+^ macrophages within tumor tissues. **E** Representative images depicting whole-slice scans for immunofluorescence in different experimental groups (magnification ×10). **F** Immunofluorescence staining using F4/80 antibodies was performed on tumor and adjacent tissue sections to identify macrophages (magnification ×200). The white dashed lines were employed to demarcate the tumor tissues from the adjacent tissues. **G** Statistical analysis were done to calculate the infiltrated macrophages. Pooled data from 3 fields (magnification ×200; *n* = 3). The Mean fluorescence intensity (MFI) of F4/80^+^ macrophages was quantified, followed by statistical analysis. (**C**, **D**, and **G**) were statistically analyzed using unpaired *t*-test. *P < 0.05, **P < 0.01, **** *P* < 0.0001.

The subcutaneous A20 cell seeded lymphoma tissue was harvested on day 16 post CD19 CAR-T treatment (Fig. 1A), and tumor size was monitored every 2 days throughout the experimental period (Fig. 1B). Compared to mice without CD19 CAR-T therapy, the group treated with CAR-T exhibited significantly reduced tumor weight, indicating successful tumor remission (Fig. 1C). The flow cytometry analysis revealed a higher proportion of tumor-infiltrating CD3^+^ T cells and CD11b^+^ myeloid cells in the CAR-T treated lymphoma mice. Notably, the CAR-T treated group exhibited a significantly elevated percentage of macrophages (F4/80^+^CD11b^+^) compared to the T cell control (Fig. 1D). To further elucidate the distribution of TAMs and explore their relationship with CAR-T treatment, the tumor along with the adjacent tissues was excised and subjected to immune fluorescent staining. The distribution of TAMs was assessed throughout the entire tumor slice. The results revealed that in the T cell control group, a majority of TAMs were localized in the tumor adjacent tissue surrounding dense tumor regions, whereas after CAR-T treatment, an increased number of TAMs infiltrated into loose tumor tissue (Fig. 1E). Further analysis depicted in Fig. 1F demonstrated a higher abundance and extensive infiltration of macrophages in the CAR-T cell treated groups compared to the T cell control, which corroborated with the flow cytometry data. Moreover, the statistical analysis revealed a conspicuous elevation in TAMs fluorescence in CAR-T treated mice, which was attributed to a higher infiltration of TAMs within the actual tumor tissues (Fig. 1G). These findings suggest active involvement of macrophages in lymphoma dynamics subsequent to CD19 CAR-T cell administration.

### The transformation of macrophages in the TME during remission and relapse status of lymphoma

**Fig. 2:**
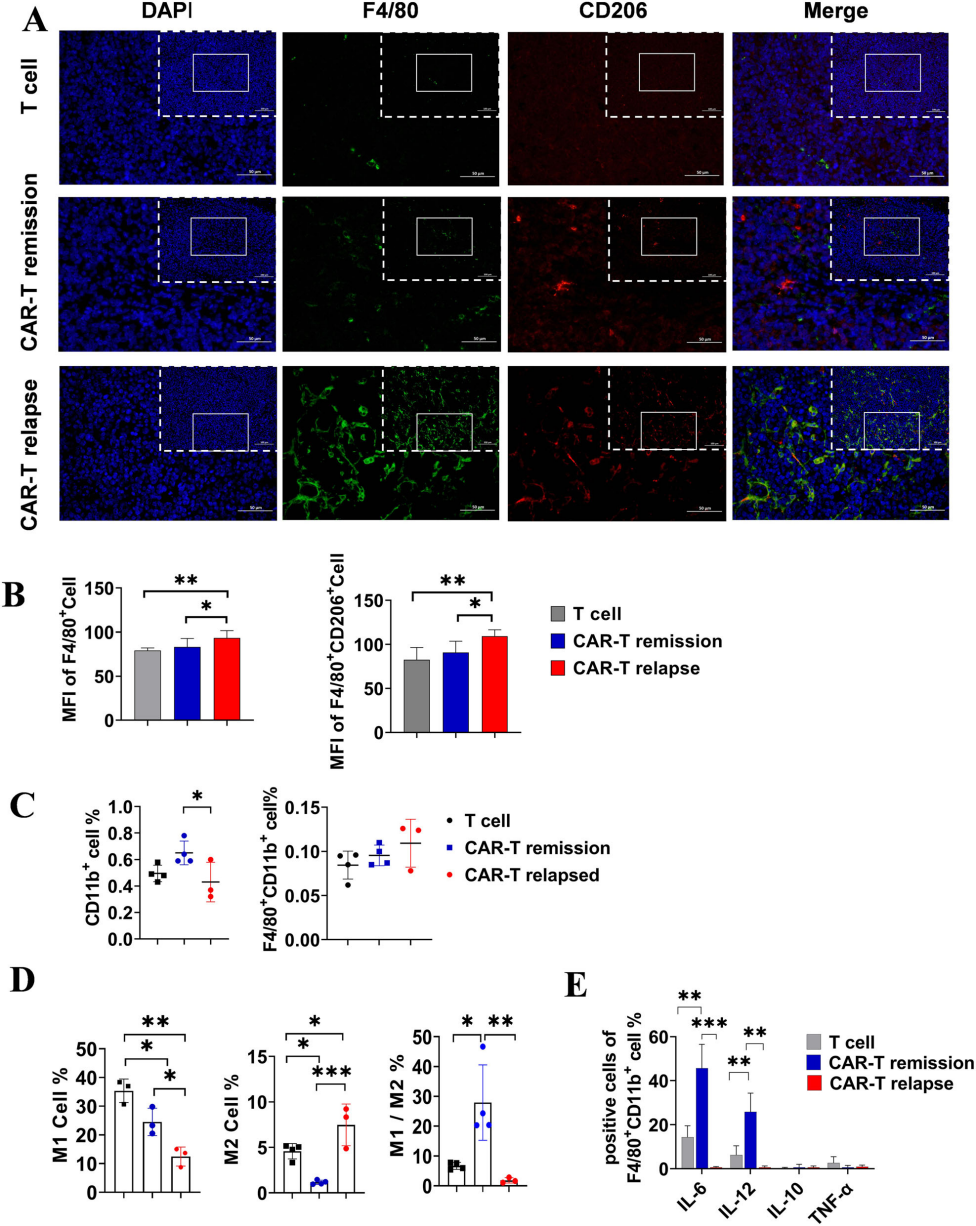
The polarization and cytokine production of macrophages in the TME following mCD19 CAR-T cell treatment in subcutaneous lymphoma under different conditions. **A** The tumor tissue sections were subjected to immunofluorescence staining using DAPI, F4/80, and CD206 antibodies for the specific labeling of macrophages. The field of view at a magnification of 400× is presented, with the solid box indicating the position of the field of view at a magnification of 200×. **B** The mean fluorescence intensity of macrophages was quantified, and subsequent statistical analysis was conducted. Pooled data from 3 fields (magnification 200×; *n* = 3) were utilized. **C** Proportions of CD11b^+^ myeloid cells and F4/80^+^ macrophages within tumor tissues. **D** Percentages and ratio changes of M1 and M2 macrophage subtypes. **E** Cytokine secretion by macrophage detected in tumor tissues. One-way ANOVA was used for statistical analysis. *P < 0.05, **P < 0.01, ***P < 0.001.

The transformation dynamics of TAMs were found to be associated with the outcomes of immunotherapy [23, 24]. In order to further analyze the characteristics of macrophages in the TME, murine models of relapsed subcutaneous lymphoma were embraced, and a combination of CD206 and F4/80 markers was employed for distinguishing M1 and M2 polarization. Compared to control and remissive mice, an increased abundance of macrophages was observed in relapsed tumor tissues subsequent to CD19 CAR-T administration (Fig. 2A). Notably, there was a significant increase in the population of CD206 and F4/80 double positive (M2) cells in relapsed mice (Fig. 2A, B). Furthermore, flow cytometry was utilized to quantify M1 and M2 cells within tumor tissues. Consistent with our previous findings indicating a decrease in total CD11b^+^ myeloid cells in the relapsed TME [19]. We observed an increasing trend of macrophages in relapsed mice, though this increase did not reach statistical significance (Fig. 2C). The proportion of M1 (F4/80^+^CD206^−^CD80^+^) was found to be lower in the relapsed group compared to the remission group, while M2 (F4/80^+^CD206^+^CD80^+^) exhibited a substantial 6.43-fold increase. Consequently, there was a significant decrease in the ratio of M1 to M2 in relapsed mice (Fig. 2D). In addition, cytokine profiling confirmed downregulations of IL-6 and IL-12 as pro-inflammatory molecules in relapsed mice (Fig. 2E). The above data illustrates the predominant pro-inflammatory role of macrophages in lymphoma-recessed mice, whereas relapsed lymphoma is associated with immunosuppressive M2 polarized TAMs.

### Efficacy of CD19 CAR-T cells in lymphoma/leukemia murine model

**Fig. 3:**
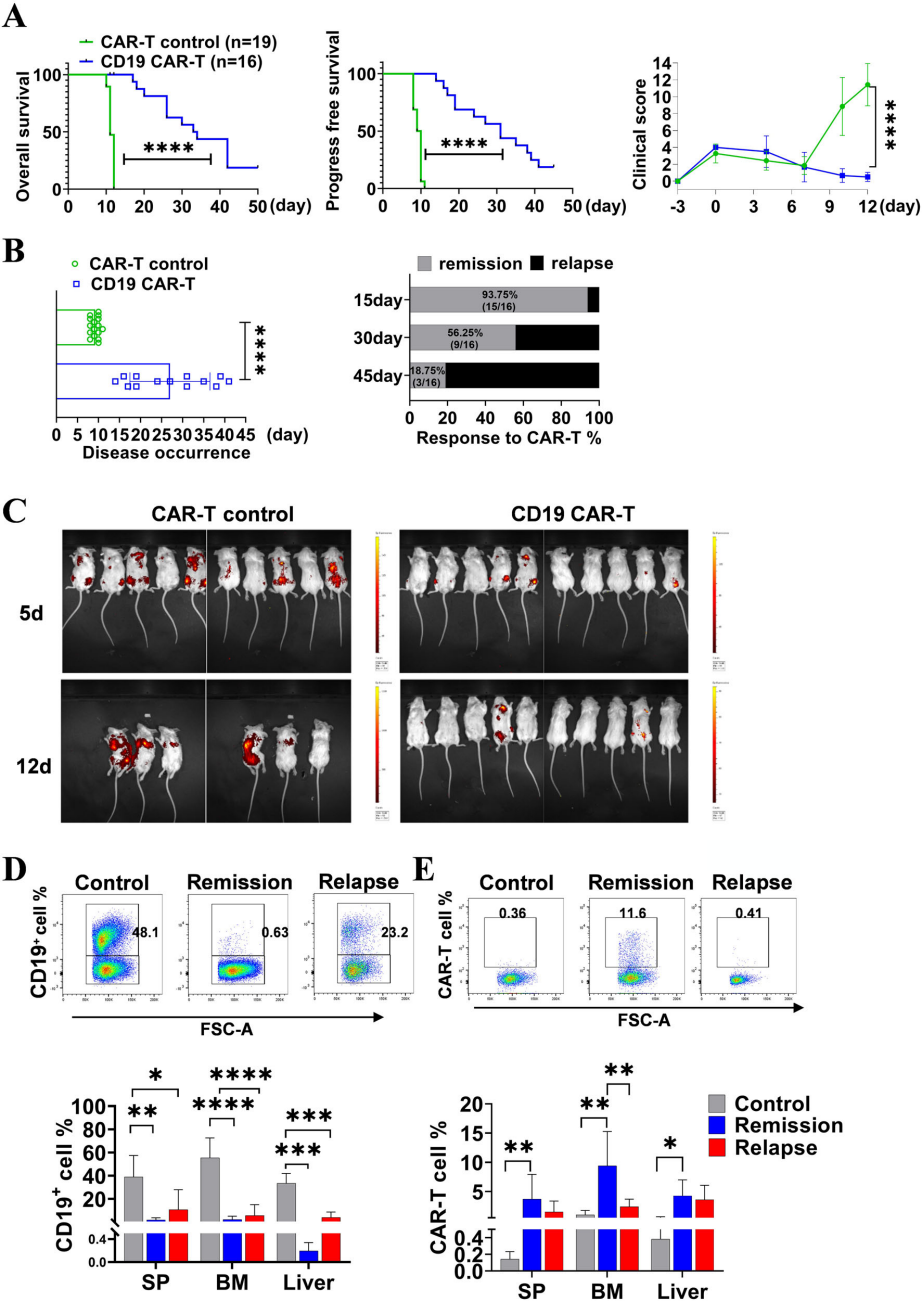
Efficacy of mCD19 CAR-T cell in murine lymphoma/leukemia model. **A** The survival of lymphoma mice following CAR-T cell treatment. The OS and PFS data were recorded from the day of CAR-T cell administration. Clinical scores were compared between mice treated with mCD19 CAR-T cells and a control group. **B** The timing of disease occurrence and response rate following anti-CD19 CAR T cell therapy was demonstrated. **C** The in vivo bioluminescent imaging of lymphoma-bearing mice injected with luciferase-expressing A20 cells and subsequently treated with CAR-T cells for 5 and 12 days. **D** The proportion of total CD19^+^ cells in BM on day 15. **E** Percentages of GFP^+^CD3^+^ CAR-T cells in BM, SP, and liver were quantified. Representative flow cytometry plots from 3 individual mice were presented. The log-rank test was employed to compare survival rates, and the *t* test was used to compare between two groups. One-way ANOVA was used for statistical analysis in (**D** and **E**). There were two independent experiments repeated. *P < 0.05, **P < 0.01, ***P < 0.001, **** *P* < 0.0001.

To imitate the aggressive progression of lymphoma, we employed a murine model of lymphoma/leukemia by intravenously injecting A20 cells into syngeneic immunocompetent BALB/c mice. This model allows for comprehensive evaluation of cellular dynamics within the remission and relapsed TME following CAR-T therapy, as previously reported [19]. In this study, we observed a significant improvement in OS and progression-free survival (PFS) with lower clinical scores in mice treated with murine CD19 CAR-T cells (Fig. 3A). While complete remission was achieved at the start of treatment, there was a progressively increasing relapse rate (56.25%) from day 30 onwards. Compared to mice without CD19 CAR-T treatment, disease occurrence was delayed (Fig. 3B). As depicted in Fig. 3C, the tumor burden demonstrated a significant reduction in luciferase-positive A20 cells upon CD19 CAR-T administration in mice. Flow cytometry analysis confirmed a lower percentage of CD19^+^ cells in remissive mice, while control group and some relapsed mice exhibited abundant CD19^+^ cells (Fig. 3D). Consistent with our previous findings, the CAR-T cells displayed robust expansion upon achieving remission but declined during relapse (Fig. 3E).

### M1 to M2 bias was related to disease procession following CD19 CAR-T therapy

**Fig. 4:**
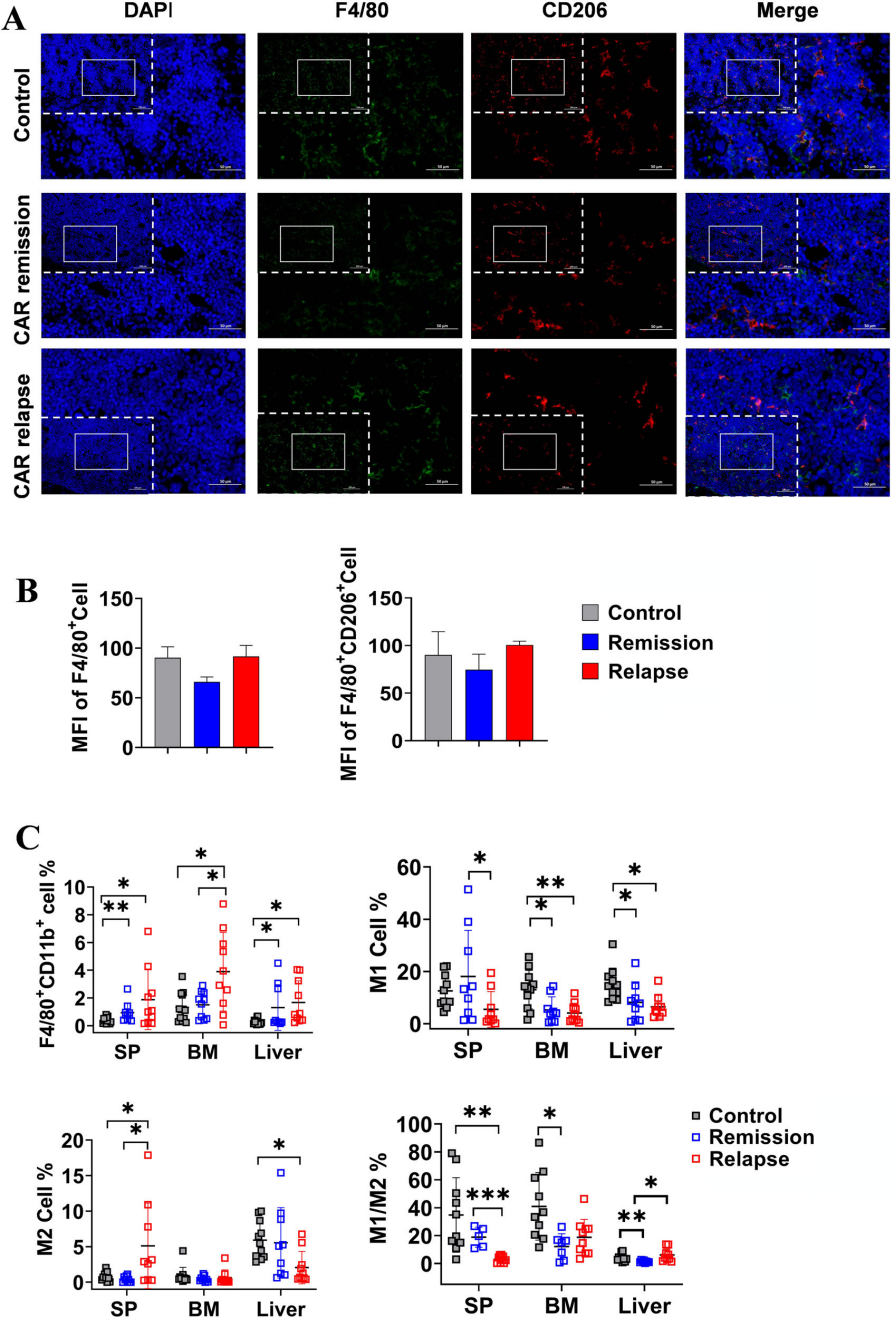
Transformation of macrophages infiltrated in the TME following mCD19 CAR-T therapy in lymphoma/leukemia mice. **A** The macrophages in the bone marrow section were stained using the F4/80 antibody. The cell nuclei were counterstained with DAPI (blue), while the macrophage markers F4/80 and CD206 were detected using HRP-conjugated goat anti-rabbit IgG and visualized with iF488-Tyramide (green) and CY3-Tyramide (red). The field of view at a magnification of 400× is presented, with the solid box indicating the position of the field of view at a magnification of 200×. **B** Statistical analysis was performed on the MFI of F4/80+ cells and CD206^+^ cells across different experimental groups. The data were pooled from 3 fields of view at a magnification of ×200, with a total sample size of *n* = 3. **C** Comparison of CD11b^+^F4/80^+^ macrophages, specifically M1 (CD11b^+^F4/80^+^CD206^-^CD80^+^) and M2 (CD11b^+^F4/80^+^CD206^+^CD80^+^) subsets, detected by flow cytometry during remission and relapse stages. (**B**, **C**) were subjected to statistical analysis via one-way ANOVA. *P < 0.05, **P < 0.01.

BM sections were stained with anti-mouse F4/80 and CD206 Abs to identify macrophage polarization. As shown in Fig. 4A, relapsed mice exhibited a stronger double fluorescence intensity of F4/80 and CD206 compared to the remission and control groups. The statistical analysis data demonstrated an increasing trend of macrophages in relapsed mice (Fig. 4B). To confirm this data, flow cytometry analysis was performed on samples obtained from different stages of mice, including SP, BM, and liver. We observed higher percentages of CD11b^+^F4/80^+^ cells upon administration of CD19 CAR-T cells in mice, with more pronounced elevation of relapsed mice (Fig. 4C). Subsequently, we identified subpopulations of macrophages and found decreased proportions of M1 macrophages in relapsed mice compared to remissive mice across SP, BM, and liver. Additionally, an increased percentage of M2 macrophages was observed in the SPs of relapsed mice along with a lower M1 to M2 ratio (Fig. 4C). Collectively, the kinetic characteristics of TAMs polarization during lymphoma progression were found to be consistent, and the M1 to M2 ratio could potentially serve as an indicator for disease staging.

### Immune activity skewing of TAMs related to the stages of lymphoma/leukemia

**Fig. 5:**
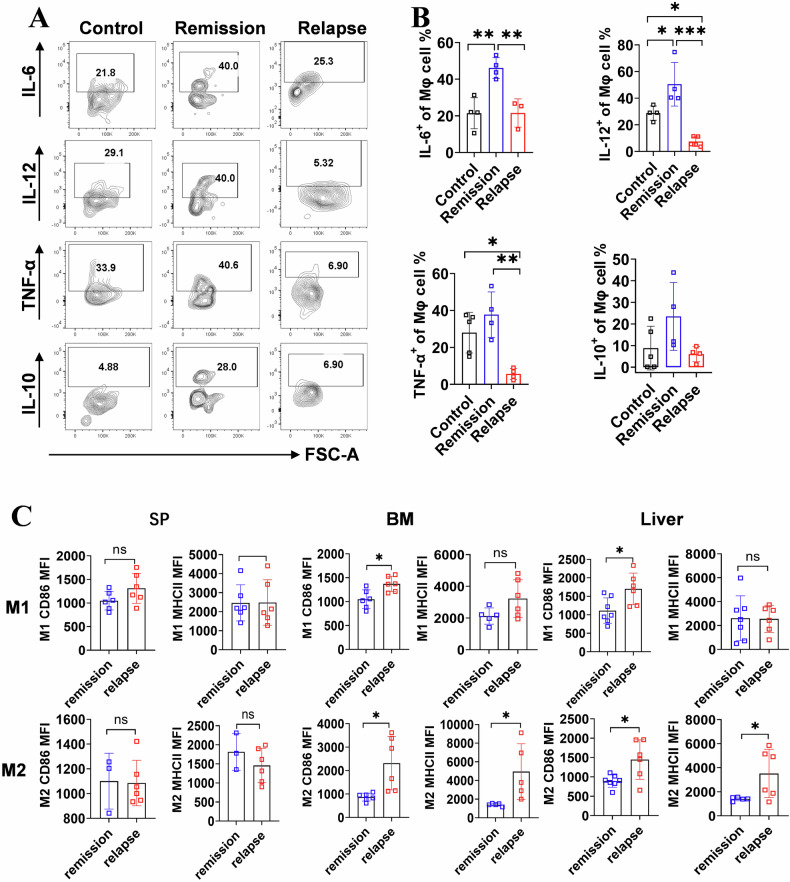
Macrophage functionality in the TME from remission to relapse following mCD19 CAR-T cell therapy. **A** Representative flow cytometry plots depicting cytokine production by macrophages from mice in remission and relapse states. **B** Statistical analysis of macrophage-derived cytokines using one-way ANOVA. **C** Expression of MHCII and CD86 on macrophage in SP, BM, and liver. Each spot indicated an individual mouse. Statistical analyses were performed using the unpaired *t*-test. *P < 0.05, **P < 0.01, ***P < 0.001.

The immune properties of TAMs correlate not only with TAM functional diversity, but also tumor prognosis [25]. Therefore, the functionality and activation of TAMs were assessed at distinct phases subsequent to CD19 CAR-T cell therapy. Remissive mice exhibited elevated levels of pro-inflammatory cytokines IL-6 and IL-12 producing TAMs, while these levels were reduced in relapsed mice. The expression of TNF-α, a tumor-related cytokine, was decreased in relapsed mice. There was no significant difference observed in the expression of the anti-inflammatory cytokine IL-10 between remissive and relapsed mice (Fig. 5A, B). Additionally, CD86 and MHCII, as active markers, were labeled on M1 and M2 types of macrophages. Only CD86 expression showed an increase on M1 cells from both BM and liver. However, compared to remissive mice, the expression of CD86 and MHCII on M2 cells from relapsed mice was significantly elevated in both BM and liver (Fig. 5C).

### M2-related mRNA enriched within the TME of relapsed mice after CD19 CAR-T therapy

**Fig. 6:**
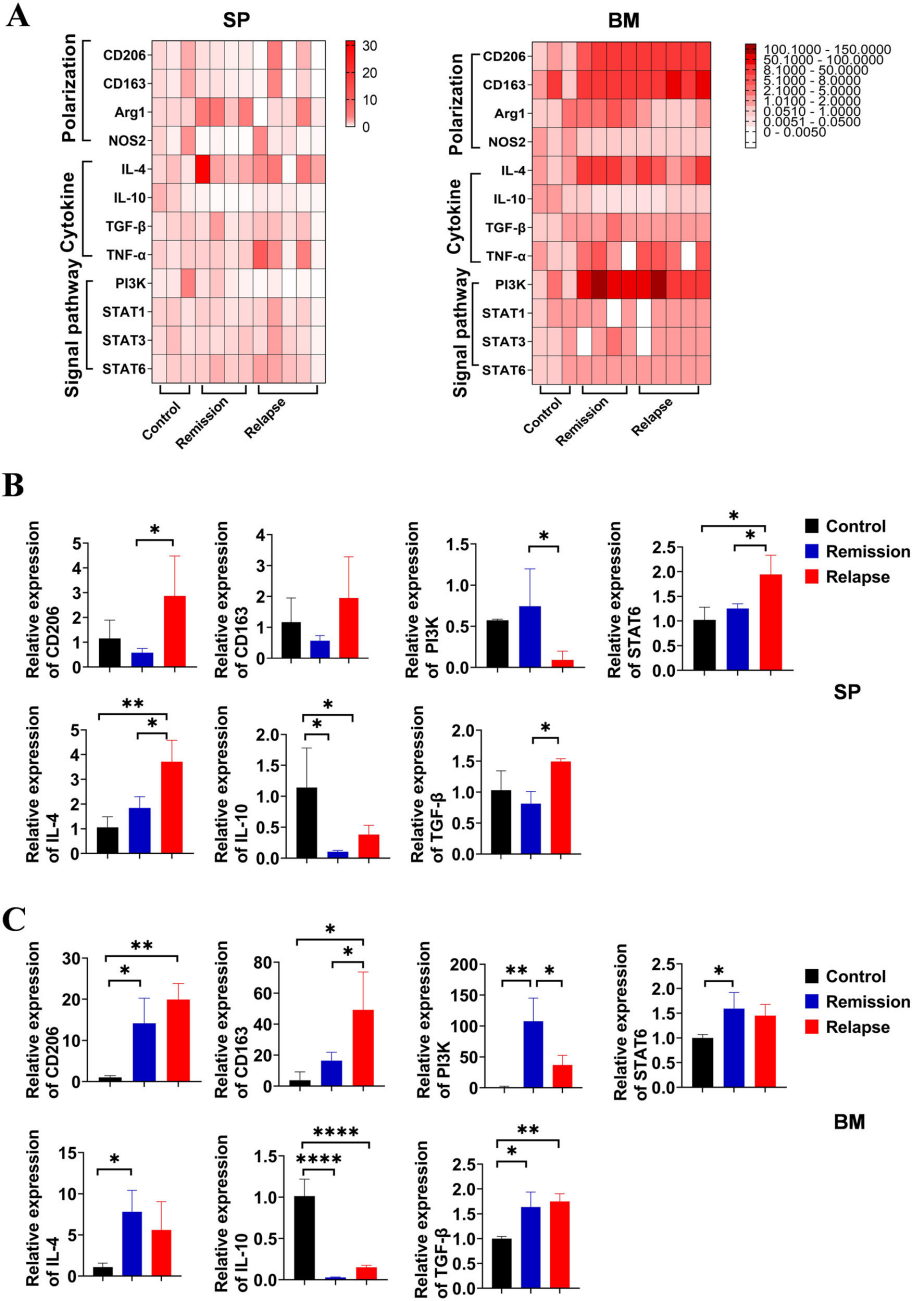
The expression of macrophage-related genes in TME from mCD19 CAR T treated lymphoma mice. **A** The relative abundance of mRNA levels detected by qRT-PCR in the SP and BM of individual mice from different experimental groups was assessed (*n* = 3–5). **B**, **C** The relative expression levels of CD206, CD163, PI3K, STAT6, IL-4, IL-10, and TGF-β, were evaluated in the SP and BM. Statistical analyses were performed using the one-way ANOVA. *P < 0.05, **P < 0.01, ***P < 0.001, **** *P* < 0.0001.

We conducted a comprehensive analysis of mRNA expression profiles associated with macrophage polarization, cytokines, and signaling pathways between remissive and relapsed mice. The heat map depicted the relative abundance of mRNA levels in SP and BM of individual mice from different experimental groups (Fig. 6A). Subsequently, a statistical analysis was conducted on the predominant mRNAs. CD206 and CD163, specifically expressed on macrophages as M2-like TAMs, exhibited higher levels in relapsed mice both in SP and BM. Activation of PI3K has been reported as an essential step towards M2 activation of macrophages [26], which showed increased expression in remissive states both in SP and BM, suggesting initiation of M2-skewing of macrophages (Fig. 6B, C). STAT6, a crucial regulator of IL-4-induced protein expression for driving macrophage M2 polarization [27, 28], exhibited increased mRNA levels in conjunction with IL-4 mRNA during relapse in mice. The IL-10 and TGF-β cytokines both utilize the STAT3 signaling pathway to induce immune suppression within the TME. However, only TGF-β exhibited increased expression in the SP of relapsed mice, while no significant differences were observed in other remissive and relapsed tissues (Fig. 6B, C). The results herein did not present data lacking statistical significance. These findings substantiate the distinctive attributes of M1 and M2 polarization phenotypes, along with the underlying regulatory pathway in response to diverse TME stimuli. Furthermore, these observations propose a potential feedback loop that contributes to lymphoma progression and subsequently influences the ultimate outcomes following administration of CD19 CAR-T cell therapy.

## Discussion

With regards to patients’ outcomes, the contribution of the TME to treatment failure in lymphoma has been emphasized [29, 30]; however, no biomarker derived from the microenvironment has demonstrated sufficient reproducibility to warrant inclusion into the routine diagnostic work-up. CAR-T cell therapy serves as a dynamic cellular intervention that establishes a closer correlation between disease progression and the evolving TME [21, 31]. Within 3 weeks following CD19 CAR-T (axi-cel) treatment, a significant upregulation of genes associated with immune cell recruitment (CCL5), immune regulation (CTLA4), and cytotoxic activity (GAMA) was observed in 14 patients with relapsed/refractory B-NHL [32]. CD19 CAR-T cell therapy elicits heterogeneous cellular dynamics, with a higher abundance of monocytes observed in non-responders compared to responders [33]. Furthermore, evidence showed that increased TAMs infiltration was negatively associated with remission status after CD19 CAR-T therapy [18]. The aforementioned information suggests that the distinct polarization status of macrophages may occur in different lymphoma stages following CAR-T therapy, and investigating this disparity could provide valuable insights for mitigating relapse.

Dissection of TAMs, using conventional and single-cell RNA sequencing (scRNA-seq) or mass cytometry by time-of-flight (CyTOF) approaches, has revealed the existence of diverse macrophage cell clusters with distinct transcriptomic and proteomic profiles [34–37]. Macrophages can now be classified into multiple distinct clusters based on distinct combinations of genes expressed. Classical M1 polarization is characterized by the upregulation of CD80 and iNOS genes, which are associated with the tumoricidal function of TAMs responsible for cancer cell engulfment and T cell recruitment. Conversely, M2 polarization has been characterized by the expression of CD206 and CD163, and is associated with an immune quiescent and immunosuppressive profile. In the present study, M2 cells were distinguished from M1 cells by their expression of CD206, while M1 cells were identified as CD80^+^CD206^−^. Single-cell-resolution approaches have been instrumental to recognize the heterogeneity of TAMs beyond the classic dichotomous classification as M1-like or M2-like. Although this concept is no longer considered appropriate, most studies continue to utilize M1–M2-associated markers for characterization of TAMs, because there is extensive experience based on the correlation between their expression and prognosis in tumor models and human cancers [38].

Gaining a comprehensive understanding of the involvement of TAMs in the disease process mediated by CAR-T is crucial for exploring novel strategies to mitigate relapse and resistance. Recently, a report has demonstrated that CAR therapy based on innate immune cells possesses the ability to eliminate TAMs, thereby enhancing the antitumor reactivity [39]. Alba et al. have discovered that TAMs expressing FRβ exhibit an M2-like phenotype within the TME. The selective elimination of these FRβ^+^ TAMs by FRβ CAR-T cells results in the recruitment of endogenous tumor-specific CD8^+^ T cells and subsequent inhibition of tumor progression [40]. Transforming TAMs by M2-polarization into the tumor-suppressive M1-phenotype is another approach for tumor therapy [23, 41]. In summary, the TAM-targeting capacity of these strategies suggest their potential for antitumor therapy that supplements cytotoxicity with remediation of TME immunosuppression and resistance following CAR-T therapy.

A more comprehensive comprehension of the function and pathways underlying polarized TAMs has established a robust foundation for the development of therapeutic strategies targeting macrophages. In our present study, higher levels of pro-inflammatory cytokines IL-6, IL-12, and TNF-α were observed in remissive mice compared to relapse mice. Although the immunosuppressive cytokine IL-10 did not show any significant differences among the groups, the substantial decrease in pro-inflammatory cytokines suggests a suppressive role of macrophages during the relapse stage of CAR-T therapy. The transformation of TAMs from an anti-tumorigenic to a pro-tumorigenic type in later stages is closely associated with the remission to relapse progression of lymphoma. In B-lymphoma cells and murine models, the muting of CREBBP/EP300 in tumor cells resulted in polarization of TAMs towards the M2 phenotype by activating the NOTCH signaling pathway and downstream CCL2/CSF1 axis [20]. Pro-inflammatory M1 macrophages rely on glycolysis whereas anti-inflammatory M2 macrophages predominantly utilize mainly fatty acid oxidation [42]. Furthermore, targeting IRE1–XBP1 and IRE1–STAT3 signaling or preserving lipid composition of the ER membrane by genetic and pharmacological approaches attenuated the pro-tumorigenic ability of TAMs and inhibited tumor progression [43]. Therefore, functional reprogramming of TAMs can be achieved with different strategies, which will contribute to acquire ideal efficacy.

In conclusion, the transformation of M1 to M2 is tightly associated with the outcomes of CD19 CAR-T therapy in lymphoma. Additionally, the ratio of M1/M2 may serve as a sensitive and reliable indicator for assessing remission or relapse status following CD19 CAR-T administration. The current data suggests that through modulation of signaling pathways or functional regulation, repolarization of TAMs based on disease progression has the potential to augment the efficacy of CAR-T therapies [44–46].

## Supplementary Information

Evaluation and design of local B cell lymphoma model

Evaluation and design of systemic B cell lymphoma/leukemia model

## Data Availability

All data relevant to the study are included in the article or uploaded as Supplementary Information.

## References

[1] Cappell KM, Kochenderfer JN. Long-term outcomes following CAR T cell therapy: what we know so far. Nature Rev Clin Oncol. 2023;20:359–71. 37055515 10.1038/s41571-023-00754-1 PMC10100620

[2] Neelapu SS, Locke FL, Bartlett NL, Lekakis LJ, Miklos DB, Jacobson CA, et al. Axicabtagene ciloleucel CAR T-cell therapy in refractory large B-cell lymphoma. N Engl J Med. 2017;377:2531–44. 29226797 10.1056/NEJMoa1707447 PMC5882485

[3] Schuster SJ, Bishop MR, Tam CS, Waller EK, Borchmann P, McGuirk JP, et al. Tisagenlecleucel in adult relapsed or refractory diffuse large B-cell lymphoma. N Engl J Med. 2019;380:45–56. 30501490 10.1056/NEJMoa1804980

[4] Abramson, Palomba JS, Gordon LI ML, Lunning MA, Wang M, Arnason J, et al. Lisocabtagene maraleucel for patients with relapsed or refractory large B-cell lymphomas (TRANSCEND NHL 001): a multicentre seamless design study. Lancet. 2020;396:839–52. 32888407 10.1016/S0140-6736(20)31366-0

[5] Wang M, Munoz J, Goy A, Locke FL, Jacobson CA, Hill BT, et al. KTE-X19 CAR T-cell therapy in relapsed or refractory mantle-cell lymphoma. N Engl J Med. 2020;382:1331–42. 32242358 10.1056/NEJMoa1914347 PMC7731441

[6] Neelapu SS, Jacobson CA, Ghobadi A, Miklos DB, Lekakis LJ, Oluwole OO, et al. Five-year follow-up of ZUMA-1 supports the curative potential of axicabtagene ciloleucel in refractory large B-cell lymphoma. Blood. 2023;141:2307–15. 36821768 10.1182/blood.2022018893 PMC10646788

[7] Locke FL, Ghobadi A, Jacobson CA, Miklos DB, Lekakis LJ, Oluwole OO, et al. Long-term safety and activity of axicabtagene ciloleucel in refractory large B-cell lymphoma (ZUMA-1): a single-arm, multicentre, phase 1-2 trial. Lancet Oncol. 2019;20:31–42. 30518502 10.1016/S1470-2045(18)30864-7 PMC6733402

[8] Chong EA, Ruella M, Schuster SJ,Lymphoma Program Investigators at the University of Pennsylvania. Five-year outcomes for refractory B-cell lymphomas with CAR T-cell therapy. N Engl J Med. 2021;384:673–4. 33596362 10.1056/NEJMc2030164

[9] Schuster SJ, Tam CS, Borchmann P, Worel N, McGuirk JP, Holte H, et al. Long-term clinical outcomes of tisagenlecleucel in patients with relapsed or refractory aggressive B-cell lymphomas (JULIET): a multicentre, open-label, single-arm, phase 2 study. Lancet Oncol. 2021;22:1403–15. 34516954 10.1016/S1470-2045(21)00375-2

[10] Zhang X, Xu K, Gale RP, Pan B. Strategies following failure of CAR-T-cell therapy in non-Hodgkin lymphoma. Bone Marrow Transpl. 2024;60:182–90. 10.1038/s41409-024-02463-4 39533016

[11] Boulch M, Cazaux M, Loe-Mie Y, Thibaut R, Corre B, Lemaitre F, et al. A cross-talk between CAR T cell subsets and the tumor microenvironment is essential for sustained cytotoxic activity. Sci Immunol. 2021;6:eabd4344. 10.1126/sciimmunol.abd4344 33771887

[12] Alizadeh D, Wong RA, Gholamin S, Maker M, Aftabizadeh M, Yang X, et al. IFNgamma is critical for CAR T cell-mediated myeloid activation and induction of endogenous immunity. Cancer Discov. 2021;11:2248–65. 33837065 10.1158/2159-8290.CD-20-1661 PMC8561746

[13] Mantovani A, Allavena P, Marchesi F, Garlanda C. Macrophages as tools and targets in cancer therapy. Nat Rev Drug Discov. 2022;21:799–820. 35974096 10.1038/s41573-022-00520-5 PMC9380983

[14] Giavridis T, van der Stegen SJC, Eyquem J, Hamieh M, Piersigilli A, Sadelain M. CAR T cell-induced cytokine release syndrome is mediated by macrophages and abated by IL-1 blockade. Nat Med. 2018;24:731–8. 29808005 10.1038/s41591-018-0041-7 PMC6410714

[15] Robinson A, Han CZ, Glass CK, Pollard JW. Monocyte regulation in homeostasis and malignancy. Trends Immunol. 2021;42:104–19. 33446416 10.1016/j.it.2020.12.001 PMC7877795

[16] Mantovani A, Marchesi F, Malesci A, Laghi L, Allavena P. Tumour-associated macrophages as treatment targets in oncology. Nat Rev Clin Oncol. 2017;14:399–416. 28117416 10.1038/nrclinonc.2016.217 PMC5480600

[17] Sica A, Larghi P, Mancino A, Rubino L, Porta C, Totaro MG, et al. Macrophage polarization in tumour progression. Semin Cancer Biol. 2008;18:349–55. 18467122 10.1016/j.semcancer.2008.03.004

[18] Yan ZX, Li L, Wang W, OuYang BS, Cheng S, Wang L, et al. Clinical efficacy and tumor microenvironment influence in a dose-escalation study of anti-CD19 chimeric antigen receptor T cells in refractory B-cell non-Hodgkin’s lymphoma. Clinical Cancer Res. 2019;25:6995–7003. 31444250 10.1158/1078-0432.CCR-19-0101

[19] Zhao K, Ren C, Tang D, Zhao L, Chen X, Wang Y, et al. The altering cellular components and function in tumor microenvironment during remissive and relapsed stages of anti-CD19 CAR T-cell treated lymphoma mice. Front Immunol. 2023;14:1101769. 36761733 10.3389/fimmu.2023.1101769 PMC9905118

[20] Huang YH, Cai K, Xu PP, Wang L, Huang CX, Fang Y, et al. CREBBP/EP300 mutations promoted tumor progression in diffuse large B-cell lymphoma through altering tumor-associated macrophage polarization via FBXW7-NOTCH-CCL2/CSF1 axis. Signal Transduct Target Ther. 2021;6:10. 33431788 10.1038/s41392-020-00437-8 PMC7801454

[21] Scholler N, Perbost R, Locke FL, Jain MD, Turcan S, Danan C, et al. Tumor immune contexture is a determinant of anti-CD19 CAR T cell efficacy in large B cell lymphoma. Nat Med. 2022;28:1872. 36038629 10.1038/s41591-022-01916-x PMC9499856

[22] Yan ZX, Dong Y, Qiao N, Zhang YL, Wu W, Zhu Y, et al. Cholesterol efflux from C1QB-expressing macrophages is associated with resistance to chimeric antigen receptor T cell therapy in primary refractory diffuse large B cell lymphoma. Nat Commun. 2024;15:5183. 38890370 10.1038/s41467-024-49495-4 PMC11189439

[23] Anderson NR, Minutolo NG, Gill S, Klichinsky M. Macrophage-based approaches for cancer immunotherapy. Cancer Res. 2021;81:1201–8. 33203697 10.1158/0008-5472.CAN-20-2990

[24] Xiang XN, Wang JG, Lu D, Xu X. Targeting tumor-associated macrophages to synergize tumor immunotherapy. Signal Transduct Target Ther. 2021;6:75. 10.1038/s41392-021-00484-9 PMC7900181 33619259

[25] Christofides A, Strauss L, Yeo A, Cao C, Charest A, Boussiotis VA. The complex role of tumor-infiltrating macrophages. Nat Immunol. 2022;23:1148–56. 35879449 10.1038/s41590-022-01267-2 PMC10754321

[26] Vergadi E, Ieronymaki E, Lyroni K, Vaporidi K, Tsatsanis C. Akt signaling pathway in macrophage activation and M1/M2 polarization. J Immunol. 2017;198:1006–14. 28115590 10.4049/jimmunol.1601515

[27] Yu T, Gan S, Zhu Q, Dai D, Li N, Wang H, et al. Modulation of M2 macrophage polarization by the crosstalk between Stat6 and Trim24. Nat Commun. 2019;10:4353. 31554795 10.1038/s41467-019-12384-2 PMC6761150

[28] Xiao J, Wang S, Chen L, Ding X, Dang Y, Han M, et al. 25-Hydroxycholesterol regulates lysosome AMP kinase activation and metabolic reprogramming to educate immunosuppressive macrophages. Immunity. 2024;57:1087–1104.e1087. 38640930 10.1016/j.immuni.2024.03.021

[29] Autio M, Leivonen SK, Bruck O, Mustjoki S, Meszaros Jorgensen J, Karjalainen-Lindsberg ML, et al. Immune cell constitution in the tumor microenvironment predicts the outcome in diffuse large B-cell lymphoma. Haematologica. 2021;106:718–29. 32079690 10.3324/haematol.2019.243626 PMC7927991

[30] Cai F, Zhang J, Gao H, Shen H. Tumor microenvironment and CAR-T cell immunotherapy in B-cell lymphoma. Eur J Haematol. 2024;112:223–35. 37706523 10.1111/ejh.14103

[31] de Visser KE, Joyce JA. The evolving tumor microenvironment: from cancer initiation to metastatic outgrowth. Cancer cell. 2023;41:374–403. 36917948 10.1016/j.ccell.2023.02.016

[32] Galon J, Rossi J, Turcan S, Danan C, Locke FL, Neelapu SS, et al. Characterization of anti-CD19 chimeric antigen receptor (CAR) T cell-mediated tumor microenvironment immune gene profile in a multicenter trial (ZUMA-1) with axicabtagene ciloleucel (axi-cel, KTE-C19). J Clin Oncol. 2017;35:3025.

[33] Haradhvala NJ, Leick MB, Maurer K, Gohil SH, Larson RC, Yao N, et al. Distinct cellular dynamics associated with response to CAR-T therapy for refractory B cell lymphoma. Nat Med. 2022;28:1848–59. 36097221 10.1038/s41591-022-01959-0 PMC9509487

[34] Puram SV, Tirosh I, Parikh AS, Patel AP, Yizhak K, Gillespie S, et al. Single-cell transcriptomic analysis of primary and metastatic tumor ecosystems in head and neck cancer. Cell. 2017;171:1611. 29198524 10.1016/j.cell.2017.10.044 PMC5878932

[35] Azizi E, Carr AJ, Plitas G, Cornish AE, Konopacki C, Prabhakaran S, et al. Single-cell map of diverse immune phenotypes in the breast tumor microenvironment. Cell. 2018;174:1293. 29961579 10.1016/j.cell.2018.05.060 PMC6348010

[36] Zilionis R, Engblom C, Pfirschke C, Savova V, Zemmour D, Saatcioglu HD, et al. Single-cell transcriptomics of human and mouse lung cancers reveals conserved myeloid populations across individuals and species. Immunity. 2019;50:1317. 30979687 10.1016/j.immuni.2019.03.009 PMC6620049

[37] Donadon M, Torzilli G, Cortese N, Soldani C, Di Tommaso L, Franceschini B, et al. Macrophage morphology correlates with single-cell diversity and prognosis in colorectal liver metastasis. J Exp Med. 2020;217:e20191847. 10.1084/jem.20191847 PMC7596819 32785653

[38] Jayasingam SD, Citartan M, Thang TH, Zin AAM, Ang KC, Ch’ng ES. Evaluating the polarization of tumor-associated macrophages into M1 and M2 phenotypes in human cancer tissue: technicalities and challenges in routine clinical practice. Front Oncol. 2020;9:1512. 10.3389/fonc.2019.01512 PMC6992653 32039007

[39] Li YR, Brown J, Yu YQ, Lee D, Zhou KY, Dunn ZS, et al. Targeting immunosuppressive tumor-associated macrophages using innate T cells for enhanced antitumor reactivity. Cancers. 2022;14:2749. 10.3390/cancers14112749 PMC9179365 35681730

[40] Rodriguez-Garcia A, Lynn RC, Poussin M, Eiva MA, Shaw LC, O’Connor RS, et al. CAR-T cell-mediated depletion of immunosuppressive tumor-associated macrophages promotes endogenous antitumor immunity and augments adoptive immunotherapy. Nat Commun. 2021;12:877. 33563975 10.1038/s41467-021-20893-2 PMC7873057

[41] Zhang JM, Zhou XY, Hao H. Macrophage phenotype-switching in cancer. Eur J Pharmacol. 2022;931:175229. 10.1016/j.ejphar.2022.175229 36002039

[42] O’Neill LAJ, Pearce EJ. Immunometabolism governs dendritic cell and macrophage function. J Exp Med. 2016;213:15–23. 26694970 10.1084/jem.20151570 PMC4710204

[43] Di Conza G, Tsai CH, Gallart-Ayala H, Yu YR, Franco F, Zaffalon L, et al. Tumor-induced reshuffling of lipid composition on the endoplasmic reticulum membrane sustains macrophage survival and pro-tumorigenic activity. Nat Immunol. 2021;22:1403. 34686867 10.1038/s41590-021-01047-4 PMC7611917

[44] Luo WC, Napoleon JV, Zhang FH, Lee YG, Wang BB, Putt KS, et al. Repolarization of tumor-infiltrating myeloid cells for augmentation of CAR T cell therapies. Front Immunol. 2022;13:816761. 10.3389/fimmu.2022.816761 PMC8889096 35250995

[45] Wang SJ, Wang JR, Chen ZQ, Luo JM, Guo W, Sun LL, et al. Targeting M2-like tumor-associated macrophages is a potential therapeutic approach to overcome antitumor drug resistance. NPJ Precis Oncol. 2024;8:31. 10.1038/s41698-024-00522-z PMC10858952 38341519

[46] van Elsas MJ, Middelburg J, Labrie C, Roelands J, Schaap G, Sluijter M, et al. Immunotherapy-activated T cells recruit and skew late-stage activated M1-like macrophages that are critical for therapeutic efficacy. Cancer Cell. 2024;42:1032–50. 10.1016/j.ccell.2024.04.011 38759656

