# Supplementary figures and images for: The transition between M1 and M2 macrophage phenotypes is associated with the disease status following CD19 CAR-T therapy for B cell lymphoma/leukemia
Source: Cell Death Dis. 2025 Apr 11;16(1):275. doi: 10.1038/s41419-025-07610-3 (PMC11992075; doi:10.1038/s41419-025-07610-3)

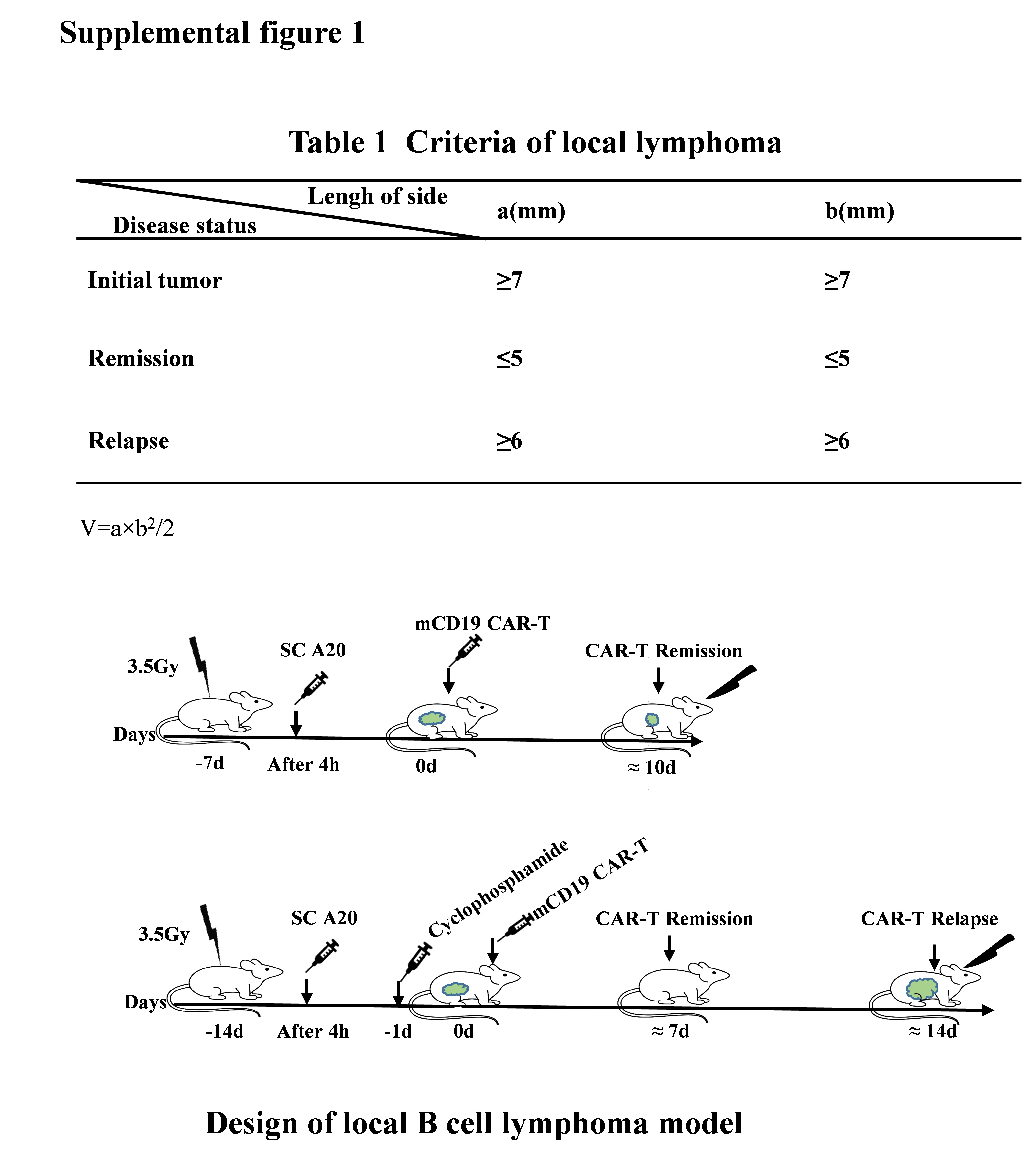

Supplement: Supplementary file 1 — Evaluation and design of local B cell lymphoma model [file 41419_2025_7610_MOESM1_ESM.jpg]

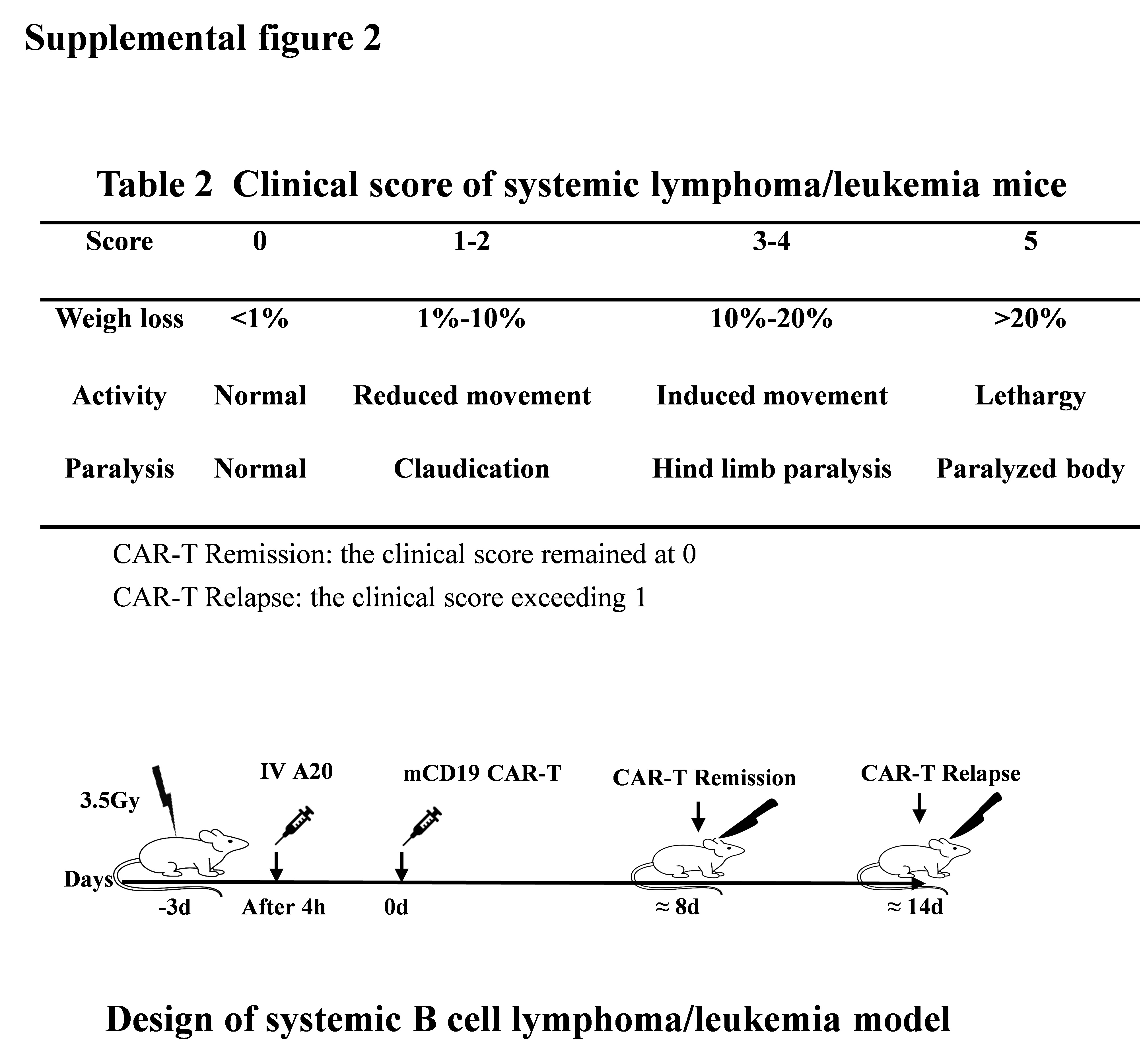

Supplement: Supplementary file 2 — Evaluation and design of systemic B cell lymphoma/leukemia model [file 41419_2025_7610_MOESM2_ESM.jpg]
